# Optimisation of Pressurised Liquid Extraction and Subsequent Hydrolysate Fermentation by *Lactiplantibacillus plantarum* for Integrated Bioprocessing of *Ulva* sp.

**DOI:** 10.3390/md23100371

**Published:** 2025-09-25

**Authors:** Aniruddh Dayanand Dave, Hakki Bilgin, Vaida Kitrytė-Syrpa, Michail Syrpas

**Affiliations:** Department of Food Science & Technology, Faculty of Chemical Technology, Kaunas University of Technology, Radvilėnų pl. 19, LT-50254 Kaunas, Lithuania; aniruddh.dave@ktu.edu (A.D.D.); hakki.bilgin@ktu.lt (H.B.); vaida.kitryte@ktu.lt (V.K.-S.)

**Keywords:** *Ulva* sp., pressurized-liquid extraction, lactic acid bacteria, fermentation, algae, marine biorefinery

## Abstract

*Ulva* sp. is a fast-growing, widely distributed marine alga with significant potential across various sectors, yet it remains underutilised. This study optimised pressurised liquid extraction (PLE) to obtain high-value fractions from *Ulva* biomass. Using a Box–Behnken design and response surface methodology, the effects of sulfuric acid concentration, temperature, and extraction time on yield, reducing sugars, total carbohydrates, and phenolic content were evaluated. Optimal conditions were identified as 110 °C, three 15 min cycles, and 3.6% sulfuric acid. Under these parameters, the extract yielded 46.9 g/100 g dry weight (DW), with 244.0 mg of reducing sugars/g DW, and 15.4 mg GAE/g DW, aligning with model predictions. The hydrolysate supported fermentation by *Lactiplantibacillus plantarum*, resulting in a growth of ~9 log CFU and the production of 3.3 g/L of lactic acid within 48 h. The antioxidant capacity remained stable post-fermentation, with CUPRAC, DPPH, and ABTS values of ~52, 38, and 18 mg TE/g DW, respectively. This work demonstrates the effectiveness of PLE in extracting valuable compounds and the feasibility of microbial fermentation of the extracts. This integrated approach highlights the potential of *Ulva* biomass and offers a platform for further applications in food, cosmetics, and nutraceuticals.

## 1. Introduction

The increasing demand for global food supplies, coupled with an excessive dependence on terrestrial agroecosystems, presents a substantial challenge to the sustainable production of food items [1]. Over the last years, social, environmental, health, and nutrition concerns have increased scientific and consumer attention to algal products and their applications in food, especially as enrichment ingredients in innovative foods [2]. In fact, research into seaweeds for food applications has gained momentum lately due to the pressing need for innovative resources that support both human health and address environmental concerns [3].

*Ulva* sp., commonly known as sea lettuce, is a green macroalgae belonging to the genus *Ulva*. They are globally distributed and frequently contribute to large-scale “green tides” in nutrient-enriched coastal waters, and have been described as tomorrow’s “wheat of the sea” in foods, as well as feeds, nutraceuticals and biomaterials [4]. *Ulva* sp. are frequently characterised by their high content of sulfated polysaccharides, particularly ulvan, which can represent up to 30% of their dry weight and is being considered for its medicinal and pharmacological applications [5,6]. In addition to ulvan, species belonging to *Ulva* contain other polysaccharides, including cellulose, glucuronan, xyloglucan, and starch, as cell wall or storage polysaccharides, with the total polysaccharide content reaching 40–50% of the biomass, making them well-suited for sugar-based bioconversions [7,8]. Interestingly, recent studies have emphasised the potential of *Ulva* sp. within integrated marine biorefineries, where it can be fractionated into multiple high-value products [9,10,11], and also for the valorisation of *Ulva* sp. for food and nutraceutical applications [12]. Towards this, enzymatic hydrolysis has been widely suggested as a method to depolymerise macroalgal polysaccharides; however, this approach often requires pretreatment, extended incubation times, and high enzyme costs [9]. Another commonly applied approach involves classical acid or base hydrolysis, which requires relatively high reagent concentrations, high amounts of solvent, and energy-intensive conditions, making the process environmentally challenging [10]. Interestingly, frequently dilute H_2_SO_4_ is preferred over HCl for acidolysis, as its higher H^+^ concentration more effectively breaks 1,3-glycosidic bonds, enhancing polysaccharide hydrolysis into monosaccharides [11].

Over the last few years, among the current green extraction technologies, pressurised liquid extraction (PLE) using elevated temperature and pressure with minimal solvent use has been suggested as an efficient and eco-friendly approach to recover valuable components from marine macroalgae, including *Ulva* sp. [12,13]. However, the efficiency and selectivity of PLE are strongly influenced by interacting factors, such as extraction temperature and time. Additionally, the H_2_SO_4_ concentration should be considered a significant parameter that also requires optimisation to increase the sugar yield further [10]. To manage this complexity, response surface methodology (RSM), particularly the Box–Behnken design, is widely used to model and optimise multiple variables efficiently with a limited number of experiments [14].

Nevertheless, the full-scale industrial exploitation of *Ulva* sp. remains relatively limited, and further studies are needed to optimise both green extraction and valorisation pathways, particularly in ways that align with the principles of a circular bioeconomy [15]. In this context, fermentation has emerged as a complementary approach within integrated algal biorefineries. Early research efforts primarily focused on converting *Ulva* into biofuels, particularly bioethanol [16] and biogas [14], as well as to a lesser extent, animal feeds and other biobased products and platform chemicals [17]. However, recent developments suggest that algal fermentation could broaden the scope of algal-derived products, thus potentially laying the ground for a novel fermentation industry of food products [18].

Despite growing interest in the biotechnological valorisation of macroalgae, no previous study has systematically optimised PLE for *Ulva* sp. using RSM and subsequently validated the fermentability of the resulting hydrolysate by a probiotic lactic acid bacteria (LAB) strain. Most existing studies involving *Ulva* sp. focus either on enzymatic saccharification or on component-specific extraction (e.g., ulvan or proteins) without subsequent fermentation of the carbohydrate-rich fraction. Additionally, while LAB have been studied in the fermentation of terrestrial biomass hydrolysates, there is limited evidence of their performance on dilute-acid extracts derived from marine macroalgae. *Lactiplantibacillus plantarum* is a well-characterised facultative heterofermentative, Gram-positive, aerotolerant probiotic LAB, known for its metabolic versatility and acid tolerance, making it a promising candidate for marine biomass valorisation. Successful growth of this strain on *Ulva* sp. hydrolysates could enable the development of functional fermented products with potential health benefits. In this study, we present a combined approach: first, optimising PLE conditions using dilute sulfuric acid to maximise fermentable sugar yield, and second, evaluating the growth and metabolic activity of *L. plantarum* on the resulting hydrolysate. By integrating green extraction and fermentation into a single workflow, we aim to establish a practical and scalable platform for sustainable macroalgal bioprocessing, thereby contributing to the broader goals of circular bioeconomy and full utilisation of marine resources.

## 2. Results and Discussion

### 2.1. Chemical Composition of Ulva sp. Biomass

**Figure 1:**
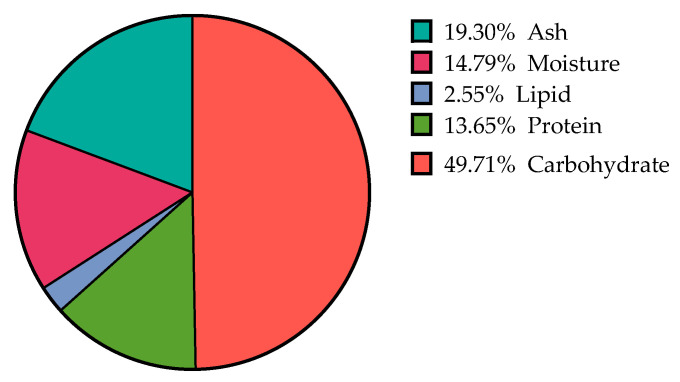
Proximate composition of the studied *Ulva* sp. biomass.

In the initial phase of this study, the chemical composition of *Ulva* sp. biomass was determined using standard methods, with the results summarised in Figure 1. As can be seen, the biomass was characterised by a high carbohydrate content (49.71%), followed by ash and proteins, while the total lipid content was low (2.55%).

The proximate composition reported in the present study was in good agreement with previous reports. In a recent review summarising the biochemical profiles of different *Ulva* species, the ash content varied between 13% and 50%, lipids ranged between 0.5% and 4%, whereas protein and total carbohydrates can range between 5% and 27% and 53% and 78%, respectively [4]. Moreover, Lee et al. have reported that dried *U. pertusa* contained 52.3% carbohydrate, 25.1% protein, 0.1% lipid, and 22.5% ash [19]. Similarly, *U. lactuca* isolated on the coast of Bangladesh showed a carbohydrate content of 39.9%, whereas the protein, moisture, and lipid contents were 20.1%, 15.3%, and 1.4%, respectively [20]. Moreover, Balar et al. reported that among 109 samples of *U. rigida*, isolated from fifteen locations along the Indian coast, the carbohydrate, protein, and lipid contents ranged from 16.6 to 65.9% DW, 4.1 to 26.0% DW, and 0.8 to 3.1% DW, respectively [21]. Overall, it is well established that, besides climatic conditions, the biochemical composition of *Ulva* species fluctuates during various growth phases [17], harvesting seasons [18], geographic locations [16], and among different species of this genus [22].

### 2.2. PLE Optimisation of Ulva sp. Biomass

#### 2.2.1. Box–Behnken Design and Response Surface Plots of PLE

PLE has been proposed as a sustainable and green technique for recovering polysaccharides and high-value components from macroalgal biomass [23,24]. Nevertheless, as with every extraction technique, several parameters are known to influence the effectiveness of PLE. In multiple cases, researchers have applied multivariate optimisation, relying on RSM to efficiently isolate various target fractions from macroalgal biomass [25,26]. In this study, to select optimal extraction conditions, the effects of three independent PLE variables, namely sulfuric acid concentration (%, *v*/*v*), temperature (T, °C), and time (τ, min), on seven response factors were evaluated using Box–Behnken design in RSM (BBD-RSM). Table 1 presents the complete experimental matrix, comprising 17 experimental runs, along with the obtained values.

**Figure 2:**
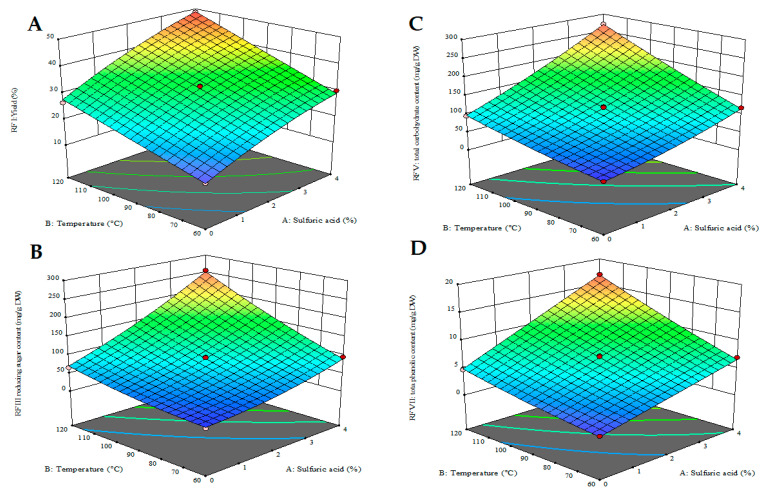
Response surface 3D plots showing the effects of PLE temperature and sulfuric acid concentration on the *Ulva* sp.: (**A**) PLE extract yield (g/100 g DW); (**B**) TRC (mg/g DW); (**C**) TCC (mg/g DW); (**D**) TPC (mg GAE/g DW).

Under the various experimental conditions, the extraction yield ranged between 13.4 and 48.7 g/100 DW (Table 1). Also, the reducing sugar content varied from 172.5 to 524.6 mg/g of extract, corresponding to 23.2 to 255.3 mg/g of DW. Moreover, the total carbohydrate content varied from 281.3 to 556 and 37.8–270.6, whereas TPC ranged between 6.57–34.91 and 0.9–17.0, expressed as mg/g of extract or DW, respectively. RSM was employed to assess both the individual and combined effects of the independent variables on the selected dependent variables, which were chosen as response factors. The interaction between pairs of independent variables and their impact on the responses is illustrated using 3D surface plots. For instance, Figure 2A illustrates how temperature and sulfuric acid concentration affect the outcome when the extraction time is held constant at 10 min per cycle (Figure 2A–D).

From the presented response surface plots, the influence of mainly temperature and sulfuric acid concentration on the extraction efficiency of key compounds from *Ulva* sp. using PLE is evident. All response variables increased with rising temperature and sulfuric acid concentration within the experimental range evaluated. As expected, increasing the sulfuric acid concentration and temperature enhances the disruption of the algal cell–matrix, thus leading to higher solubilisation and recovery of intracellular components. Notably, the most significant increases are observed in the reducing sugar and total carbohydrate content, indicating efficient hydrolysis of polysaccharides into fermentable sugars under elevated thermal and acidic conditions. ANOVA results further supported these observations, with the summarised results for all models presented in the Supplementary Materials (Table S1). BBD-RSM models were modified by removing the non-significant (*p* > 0.05) linear and quadratic effects of the independent variables in order to improve their clarity and predictive accuracy. Based on these results, all reduced quadratic models were statistically significant (*p* < 0.05), with F-values ranging from 138.17 to 656.79 for RFI and RFVI, respectively (Supplementary Materials, Table S1). Additionally, all models exhibited good reproducibility, as indicated by low coefficients of variation (2.6–6.4%), high R^2^ values (>0.98), and minor differences between adjusted and predicted R^2^ values (<0.20). Overall, these indicators confirm that the models reliably fit the experimental data (Supplementary Materials, Table S2). The final equations describing the polynomial models (in actual factors) of RFs within the determined experimental range are reported in the Supplementary Materials (Equations (S1)–(S7)).

As indicated by the ANOVA data, the linear functions of sulfuric acid and temperature were the primary extraction parameters responsible for the observed changes in the total extract yield (Supplementary Materials, Table S1, RFI). They also contributed to the changes in the other studied RFs (Supplementary Materials, Table S1, RFII–RFVII). As an example, the significance of the functions for the RFIII (total reducing sugar content, mg per g of DW), decreased as follows: sulfuric acid concentration < temperature < linear interaction of temperature and sulfuric acid concentration < linear function of time < quadratic effect of sulfuric acid concentration < linear interactions between time and sulfuric acid, or temperature < quadratic effect of temperature (Supplementary Materials, Table S1, RFIII). This observation is also supported by the Pareto charts (Supplementary Materials, Figure S1A–G), indicating that sulfuric acid concentration and PLE temperature are individually responsible for 40–50% and 30–35% of the observed changes in various RFs, respectively. At the same time, the cumulative effects of extraction time, as well as the linear and quadratic interactions of the independent variables, are less important in these models.

#### 2.2.2. Simultaneous Response Optimisation and Model Validation

In this section of the study, PLE of *Ulva* sp. was optimised using numerical optimisation and the desirability function within a defined range of variables. Within the Design-Expert software, multiple responses and factors were integrated into a single desirability function. Multi-response optimisation was then applied to identify the conditions that maximise overall desirability (>90% of the response factor values within the experimental range), subject to the specified constraints of independent variables. Namely, PLE time was designated as “in range”, while the two most influential independent variables—extraction temperature and sulfuric acid concentration—were set as “minimise”. The latter was chosen to reduce overall production costs and limit the formation of Maillard reaction products, which could negatively impact the subsequent fermentation process. Using these criteria, the suggested optimal extraction conditions were a temperature of 110 °C, an extraction time of 15 min per cycle, and a sulfuric acid concentration of 3.6% (Table 2). To assess the model’s predictive accuracy, triplicate extractions were conducted under the identified optimal PLE conditions. The predicted and experimental values are summarised in Table 2. The experimental results for all response factors closely aligned with the predicted values, showing only minor deviations (Table 2). Specifically, under the optimised conditions, the extract yielded 46.9 g/100 g DW, with 244.0 mg/g DW of total reducing sugars, 260.3 mg/g DW of total carbohydrates, and 15.4 mg GAE/g DW of TPC, which were consistent with the model’s predictions (Table 2).

The observed values in this study are substantially higher, especially regarding extraction yield, than those reported in previous studies utilising PLE for *Ulva* sp. For instance, Keramane et al., using PLE with an ethanol-water (75:25) mixture at 120 °C and 1500 psi to extract *U. intestinalis* and *U. lactuca* biomass, reported that the first strain showed the highest extraction yield (6.5%) among the species, while both *Ulva* species exhibited low phenolic content and antioxidant capacity [23]. In another study with *U. lactuca*, where PLE was performed at 50 °C with 100% ethanol and 1500 psi, the authors reported a very low extraction yield (0.23%) and a low total phenolic content [27]. Furthermore, Tierney et al. compared PLE conditions for *U. intestinalis* using water, an ethanol/water mixture (80:20), and an acetone/water mixture (80:20) at varying temperatures and pressures. In their case, while water PLE yielded the highest extraction (19.3%), the aqueous acetone resulted in the highest phenolic content for *U. intestinalis* [28]. Nevertheless, it should be noted that the use of 4% sulfuric acid in this study represents a notable difference from the solvent systems explored in the aforementioned reports for *Ulva* sp. using PLE. Moreover, the efficiency of PLE is evident when comparing these values with those of previous studies that have also employed dilute acids as a pretreatment for saccharification prior to fermentation. Specifically, in a previous report where the optimisation of dilute acid hydrolysis of *U. lactuca* was performed, the authors reported that 0.5 N H_2_SO_4_ with a 5% *w*/*w* substrate concentration at 121 °C were the best condition for the autoclave treatment. When these conditions were applied prior to enzymatic treatment, the biomass yielded 214.6 mg/g DW of reducing sugars [29]. In another study, the pre-heat treatment (120 °C, 1 h) of *U. fasciata* biomass was followed by enzymatic hydrolysis for 36 h at 45 °C, resulting in a maximum sugar yield of ~207 mg/g DW [29]. The same authors reported that for the dilute acid treatment alone, the yield ranged from 28 to 114 mg/g DW [30]. Lastly, in a report where *U. pertusa* underwent high thermal liquefaction at 400 °C and 40 MPa, the authors reported a reducing sugar yield of 352 mg/g DW [31].

### 2.3. Fermentation of Ulva sp. Hydrolysate Obtained by PLE

#### 2.3.1. Growth Kinetics and pH Changes

**Figure 3:**
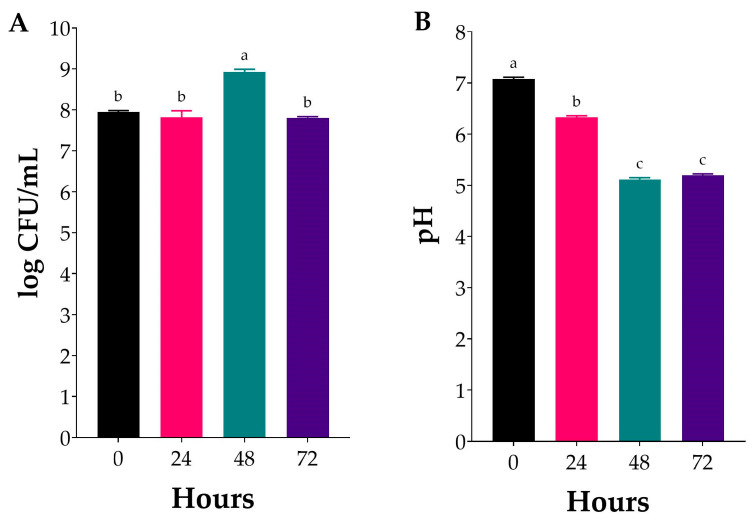
Bacterial growth (**A**) and pH changes (**B**) during fermentation of *Ulva* sp. hydrolysate. Values are expressed as mean ± standard deviation (*n* = 3). Different lowercase letters indicate significant differences (one-way ANOVA, *p* < 0.05).

Following the validation of the PLE models, the extract obtained under optimal conditions was subjected to fermentation by *L. plantarum* for 72 h, with the results summarised in Figure 3. The obtained PLE extract was used as the sole carbon source for microbial fermentation by the chosen strain. The viable cell count remained stable during the first 24 h at ~8 log CFU/mL, and then substantially increased, reaching ~9 log CFU/mL after 48 h, after which it further declined (Figure 3A). During the first 24 h of fermentation, cell growth was slow, indicating a lag phase. This delay may be attributed to the use of MRS medium for inoculum preparation rather than seaweed hydrolysate, which could have promoted better acclimatisation of *L. plantarum* to the fermentation substrate, as previously reported [32]. Moreover, the decline post 48 h is likely due to a combination of interacting factors. Firstly, substrate depletion, and particularly the fermentable carbohydrate content after 48 h, which are key nutrients for the continued growth and metabolism of microorganisms. Considering that the PLE extract was the sole carbon source provided for fermentation, this is highly likely, especially given the anticipated complexity and heterogeneity of the monosaccharide composition of the obtained extract. Moreover, the accumulation of organic acids, resulting from the conversion of fermentable carbohydrates primarily to lactic acid, is a well-documented growth-limiting factor in LAB fermentations. pH values between 5.6 and 6.0 are considered optimal for the growth of LAB during production and storage [33]. Indeed, as expected, L. plantarum growth was accompanied by a progressive change in the pH values of the fermentates (Figure 3B). Specifically, the pH level decreased from 7.0 to 6.3 within 24 h, and further dropped to 5.1 at 48 h, remaining stable thereafter (Figure 3B). Overall, these observations indicate that after 48 h, reduced fermentable carbohydrate concentrations or strong buffering in the extract further limit the growth and acidification of the medium.

Although many studies do not report the growth of the fermenting microorganism, these findings are consistent with previous reports that demonstrate the fermentability of *Ulva*-derived sugars and the subsequent production of lactic acid [34,35,36,37,38]. Interestingly, one previous report has shown that *Ulva* sp. hydrolysates cannot be readily fermented by LAB, thus indicating a potential challenge and the need for further optimisation in fermentation strategies [39]. These studies strongly suggest that various factors, such as the hydrolysis method, strain of LAB used, or composition of the obtained hydrolysates, can significantly influence fermentability outcomes. It is worth noting that, in addition to the observed microbial growth (Figure 3A), the feasibility of fermenting *Ulva* sp. extracts may have significant implications for the development of food and nutraceutical products. The ability of *L. plantarum* to reach high levels (>10^8^ CFU/mL) in this study demonstrates its potential for developing functional products, such as probiotic beverages, fermented seaweed condiments, or dried powders. Moreover, the mild acidification observed could enhance flavour by reducing marine off-notes and introducing a tangy, lactic character, thereby improving consumer acceptability, as previously indicated in algal fermentation studies [40].

#### 2.3.2. Consumption of Monosaccharides and Production of Organic Acids During Fermentation of *Ulva* sp. Hydrolysate

**Figure 4:**
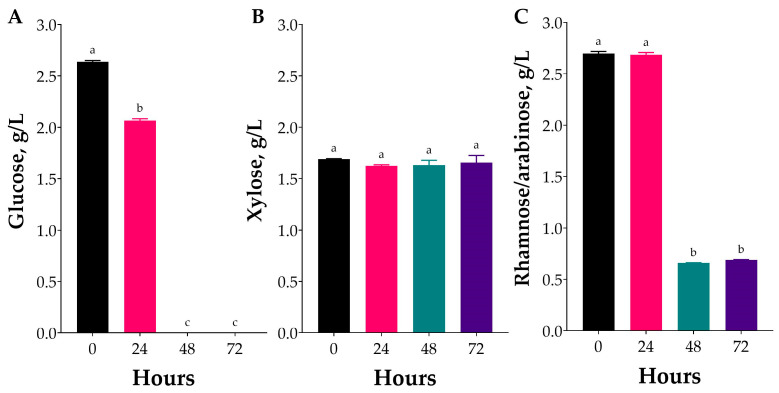
Changes in glucose (**A**), xylose (**B**) and rhamnose/arabinose (**C**) contents during the fermentation of *Ulva* sp. hydrolysate. Values are expressed as mean ± standard deviation (*n* = 3). Different lowercase letters indicate significant differences (one-way ANOVA, *p* < 0.05).

The specific carbohydrate fermentation pathways utilised by a bacterial strain determine the types of organic acids it produces. In LAB, sugar metabolism is tightly controlled through both a global regulatory repressor and sugar-specific regulatory mechanisms within operons [41]. The main sugars found in hydrolysates from green algae include rhamnose, glucose, arabinose, xylose, and uronic acids [42]. In this study, chromatographic analyses revealed that the PLE extract, used as the fermentation substrate, was primarily composed of glucose and rhamnose, with smaller amounts of xylose and arabinose (Figure 4A–C).

**Figure 5:**
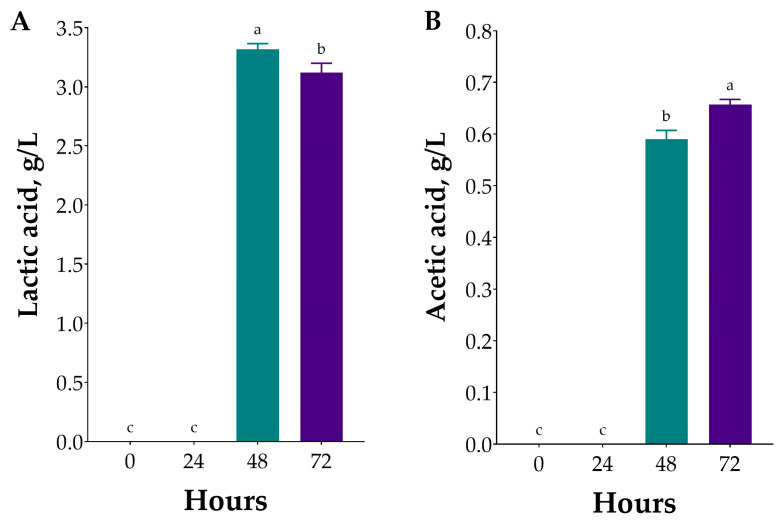
Changes in lactic acid (**A**) and acetic acid (**B**) contents during the fermentation of *Ulva* sp. hydrolysate. Values are expressed as mean ± standard deviation (*n* = 3). Different lowercase letters indicate significant differences (one-way ANOVA, *p* < 0.05).

Although the chromatographic method does not allow for the resolution of rhamnose and arabinose, it can be reasonably inferred that rhamnose predominates, given its release through ulvan hydrolysis and its role as the principal monosaccharide in this polysaccharide of *Ulva* sp. The chromatographic data further support this inference. At the initiation of the experiment, the concentrations of glucose and rhamnose/arabinose were 2.6 g/L and 2.7 g/L, respectively, while xylose was detected at approximately 1.6 g/L. Similar sugar profiles, dominated by glucose and rhamnose, have been previously reported in *Ulva* sp. biomass subjected to thermochemical hydrolysis [43] or subcritical water hydrolysis [44]. As depicted in Figure 4, glucose content was reduced by 24 h, and was completely consumed within 48 h (Figure 4A). Similar observations can be made for rhamnose/arabinose, where it can be hypothesised that rhamnose was partially consumed at 24 h and was entirely consumed within this timeframe, with the residual content (~0.6 g/L) ascribed to arabinose (Figure 4C). Similarly, xylose levels remained unchanged (Figure 4B), indicating that the strain used may not metabolise arabinose or xylose under the given conditions. Several *L. plantarum* strains demonstrate metabolic flexibility in nutrient-rich environments, utilising multiple sugars simultaneously; however, they typically show a strong preference for glucose, while the ability to catabolise arabinose and rhamnose is strain-dependent [45]. Wild-type strains of *L. plantarum* isolated from various niches generally do not have genes related to xylose utilisation [46]. As depicted in Figure 4 and Figure 5, during the first 24 h of fermentation, carbohydrate consumption and metabolite production were small, and compatible with the observed lag phase during the microorganism’s growth. Also, the depletion of glucose and rhamnose after 48 h and the inability of this strain to catabolise xylose may further support the previous hypothesis, which linked nutrient depletion to the observed decline in the growth of *L. plantarum*.

Monosaccharide consumption coincided with a drop in pH from neutral to 5.1 within 48 h, as reported in the previous section, indicating active acid production. In this study, the production of organic acids, specifically lactic and acetic acids, during fermentation was monitored using HPLC, with the results summarised in Figure 5. The analyses showed that lactic acid was detectable within 48 h of fermentation, after which its levels declined (Figure 5A). The concentration of lactic acid in PLE hydrolysates fermented with *L. plantarum* reached its peak (~3.3 g/L) at 48 h of fermentation, corresponding with the observed pH decline discussed earlier. On the other hand, acetic acid production occurred at 48 h, after which it increased, reaching its maximum (~0.6 g/L) at the last checkpoint at 72 h (Figure 5B). The production of acetic acid is expected, as *L. plantarum* strains that contain the rhamnose operon typically convert this deoxy sugar into intermediates that enter the pentose phosphate pathway, which frequently leads to heterofermentative metabolism, where rhamnose catabolism produces both lactic acid and acetic acid [45].

The observed values correspond to organic acid production equivalent to ~0.8 g of acids per g of utilised fermentable sugars, or 0.11 g per g of *Ulva* sp. biomass. These findings are similar to previous reports utilising *L. plantarum* or other LAB for lactic acid production from *Ulva* biomass. Specifically, in the first report on the utilisation of seaweeds for lactic acid and ethanol production, Uchida and Murata reported values up to 0.13 g of lactic acid per g of *Ulva* sp. biomass [47]. Moreover, Helmes et al. reported that *Ulva* sp. hydrolysates yielded 0.9 g of lactic acid per gram of fermentable saccharides, with *L. plantarum* as the fermenting microorganism [34]. In other reports, the observed lactic acid yields were higher, reaching 0.2 and 0.4 g of lactic acid per g of *U. pertusa* [48] and *U. fasciata*, respectively [36]. Overall, typical lactic acid titers from *Ulva* sp. fermentations vary widely depending on pretreatment and microbial strain. Titers in the range of 3–5 g/L are common for batch fermentations using LAB, with optimised processes reaching up to 7–10 g/L under fed-batch or co-culture conditions [32]. Although the lactic acid concentration achieved in this study (~3.3 g/L) may seem modest, it should be viewed in light of the study’s primary goal to demonstrate process feasibility rather than maximising lactate yield. Additionally, the PLE hydrolysate used in this study served as the sole carbon source without any further nutrient supplementation. The limited fermentable sugar content and the inability of this strain to utilise xylose restrict further acid production.

#### 2.3.3. Changes in the In Vitro Antioxidant Capacity During Fermentation of *Ulva* sp. Hydrolysate

Ulvan and other *Ulva* sp. derived fractions, including acidic extracts, have garnered scientific attention due to their antioxidant properties and potential applications in both food and cosmetic products [49]. Moreover, a previous review has highlighted the potential of fermentation to alter the antioxidant capacity of microalgae and the antioxidant properties of brown and red macroalgae. However, information on the effect of fermentation on the antioxidant capacity of green macroalgae, or their extracts, is scarce [50]. In this section of the study, the influence of fermentation on the in vitro antioxidant capacity of the obtained hydrolysate was evaluated. To achieve this, three distinct and commonly used assays, namely, the ABTS, DPPH radical scavenging, and cupric CUPRAC assays, were utilised, with the results summarised in Table 3.

A common observation in all three assays was a slight increase in antioxidant capacity values, observed after 48 h of fermentation. Specifically, the TEAC values shifted from 49.3 to 52.5, 36.9 to 38.5, and 17.1 to 18.0 mg TE/g DW for the CUPRAC, DPPH, and ABTS assays, respectively (Table 3). However, with the exception of the DPPH assay, it is worth noting that this effect was not statistically significant (*p* > 0.05). The antioxidant capacity of aqueous *Ulva* sp. extracts is frequently attributed to its polysaccharides, phenolics and pigments [51]. While increases in antioxidant capacity during fermentation are related to the release of bound phenolics or bioactive peptides from the plant or algal matrix used, this mechanism is unlikely here, as the extract, rather than the whole *Ulva* sp. biomass, was used as the fermentation substrate. Nonetheless, the obtained PLE extract showed notable in vitro antioxidant capacity, which was maintained after the fermentation process. From a product development perspective, the observed stability can be considered an advantage, as it demonstrates that fermentation can enhance microbial safety and deliver functionality without compromising the inherent extrinsic properties. As a future step, though, utilisation of the entire treated biomass would be an interesting approach to evaluate whether fermentation may enhance the antioxidant activity due to the aforementioned mechanisms. Overall, the obtained PLE extract, whether before or after fermentation, shows potential for applications in the food or nutraceutical industries.

## 3. Materials and Methods

### 3.1. Ulva sp. Biomass and Reagents

Dry powder of *Ulva* sp. biomass was purchased from Aqualgae (Aqualgae S.L., A Coruña, Spain) and was stored in the dark at room temperature until further analyses.

2,2′-Azino-bis(3-ethylbenzothiazoline-6-sulfonic acid) diammonium salt (ABTS), Sigma-Aldrich, Steinheim, Germany), 2,2-diphenyl-1-picrylhydrazyl hydrate (DPPH^•^, 95%), 3,4,5-trihydroxybenzoic acid (gallic acid, 99%, Sigma-Aldrich, Steinheim, Germany), 2-(3-hydroxy-6-oxo-xanthen-9-yl)benzoic acid (fluorescein (FL), Fluka Analytical, Bornem, Belgium), 6-hydroxy-2,5,7,8-tetramethylchroman-2-carboxylic acid (Trolox, 97%, Sigma-Aldrich, Steinheim, Germany), Folin & Ciocalteu’s phenol reagent [2 M], Fluka Analytical, Bornem, Belgium), NaCl, KCl, KH_2_PO_4_, K_2_S_2_O_8_ (Lach-Ner, Brno, Czech Republic), Na_2_HPO_4_ (Merck KGaA, Darmstadt, Germany), Na_2_CO_3_ (Sigma-Aldrich), H_2_SO_4_, NaOH, H_3_PO_4_, (Sigma-Aldrich), HCl (35–38%, Chempur, Piekary Śląskie, Poland), acetonitrile, methanol, dichloromethane, hexane (HPLC grade, Sigma-Aldrich Chemie, Steinheim, Germany), catalytic tablet (K_2_SO_4_, CuSO_4_, Sigma-Aldrich), ASE filters (Glass Fibre-(X)-Cellulose, Dionex Corporation, Sunnyvale, CA, USA), diatomaceous earth (100% SiO_2_, Dionex Corporation, Sunnyvale, CA, USA), cotton-wool (Belfast-cotton, Poland). For extractions, concentrated H_2_SO_4_ (98%) (Chempur, Piekary Śląskie, Poland) was used. Analytical standards of lactic acid, acetic acid, glucose, rhamnose, arabinose, and xylose were purchased from Sigma-Aldrich (Sigma Aldrich, Steinheim, Germany). All solvents used for extraction and chromatographic analysis were of analytical and HPLC-grade, respectively.

### 3.2. Proximate Composition of Ulva sp. Biomass

Protein content was determined using the Kjeldahl method (AOAC 960.52). Moisture and ash contents were determined gravimetrically (AOAC 925.10 and 900.02, respectively). The lipid content was assessed after Soxhlet extraction with n-hexane, as previously reported by our research group [52].

### 3.3. PLE of Ulva sp. Biomass by Experimental Design

PLE was performed using 1.2 ± 0.001 g of *Ulva* sp. biomass, mixed with an equal amount of diatomaceous earth and loaded into stainless-steel extraction cells. The cells were equipped with cellulose filters fitted at both ends. The extractions were carried out in a random order using an ASE-350 system (Thermo Scientific Dionex, Sunnyvale, CA, USA) under a consistent pressure of 10.3 MPa. The system was pre-heated for 5 min, followed by a full-volume cell flush and a nitrogen purge lasting 120 s. The collected samples were then neutralised (pH = 7) with NaOH. Neutralised samples were then frozen with liquid nitrogen and subsequently freeze-dried.

Optimisation of PLE conditions was performed using a BBD-RSM to evaluate the effect of the selected independent variables, namely extraction temperature, time and sulfuric acid concentration, on the chosen response factors (RFI-RFVII). The complete design consisted of 17 experimental runs, including 5 centre points, developed using Design-Expert software version 12.0.8.0 (Stat–Ease Inc., Minneapolis, MN, USA). All PLE extraction experiments were performed in random order and in triplicate.

### 3.4. Determination of Reducing Sugar Content

The total reducing sugar content (TRC) was assessed using the 3,5-dinitrosalicylic acid (DNS) assay, as previously described by Miller [53] with modifications suggested by Teixeira et al. [54]. Briefly, the DNS solution was prepared by dissolving 1 g of 3,5-dinitrosalicylic acid reagent and 30 g of sodium potassium tartrate in 100 mL of 0.4 M NaOH. Then, 1 mL of the sample was mixed with an equal volume of the DNS solution, and the samples were vortexed for 30 s. Afterwards, the samples were placed in a boiling water bath for 5 min and then allowed to cool to room temperature. Following this, 6 mL of distilled water was added, and the absorbance of each sample was determined at 540 nm using a GENESYS 150 UV–Vis spectrophotometer (Thermo Fisher Scientific, Waltham, MA, USA). Values were expressed as glucose equivalents, using an external calibration curve (0.2–1 mg mL).

### 3.5. Determination of Total Carbohydrate Content

The total carbohydrate content (TCC) was determined using the sulfuric acid-UV method, as reported by Albalasmeh et al., with slight modifications [55]. Specifically, a 5 mg/mL *Ulva* sp. extract solution was diluted with distilled water, and then 3 mL of concentrated sulfuric acid was rapidly added to the test tube. The samples were then vortexed for 30 s. The solution was then immediately cooled by placing the vial in an ice bath for 2 min. Lastly, the UV absorption was measured at 315 nm using a UV-Vis spectrophotometer (T60, PG Instruments Ltd., Wibtoft, England).

### 3.6. Determination of Total Phenolic Content

The total phenolic content (TPC) was estimated using the Folin–Ciocalteu’s method as previously described by our research group elsewhere [56]. Briefly, 150 µL of sample or blank solution was added to 750 µL of Folin–Ciocalteu’s reagent, and the samples were incubated for 3 min. Afterwards, 600 μL of a 7.5% Na_2_CO_3_ solution was added, and the samples were incubated for 2 h at room temperature. Lastly, the absorbance of the optically clear supernatant was measured at 760 nm against a reagent blank using a UV-Vis spectrophotometer (T60, PG Instruments Ltd., Wibtoft, England). TPC was expressed as gallic acid equivalents (mg GAE/g sample), using a dose–response curve for gallic acid.

### 3.7. Fermentation of Ulva sp.Hydrolysate

#### 3.7.1. Bacterial Strain and Media Preparation

The MRS media was prepared by dissolving 14.04 ± 0.001 g in 200 mL of distilled water, followed by autoclaving at 121 °C for 15 min. *Lactiplantibacillus plantarum* subsp. *plantarum* DSM 24624 were purchased from UAB Biometrija (Kaunas, Lithuania). Before fermentation, *L. plantarum* DSM 24624 was activated by two passages on MRS broth medium (De Man, Rogosa, and Sharpe, Merck, Germany). The bacterial inoculum was cultured in 10 mL of MRS broth at 37 °C for 24 h before inoculation.

#### 3.7.2. Fermentation of PLE Extract with Lactic Acid Bacteria

Fermentation experiments were conducted in triplicate using hydrolysates obtained under optimal conditions. Specifically, for each replicate 1.2 g ± 0.001 of *Ulva* sp. biomass was extracted at 110 °C for three 15 min cycles using 3.6% sulfuric acid. Prior to fermentation, PLE extracts from technical replicates were combined and neutralised to pH 7.0 using NaOH to eliminate residual sulfuric acid. The final volume was then adjusted with sterile water to 120 mL. Further, 40 mL were then transferred to a 50 mL Duran flask in three technical replicates, individually, under sterile conditions. The samples were then inoculated with *Lactiplantibacillus plantarum* DSM 24624 (7.9 log CFU/mL), and immediately placed inside a Thermo Scientific™ Oxoid™ AnaeroJar™ (Thermo Fisher Scientific Inc., Waltham, MA, USA) anaerostats to maintain anaerobic conditions using Oxoid™ AnaeroGen™ Compact Sachet, and were maintained at 37 °C. Microbial plating and pH measurements were performed at 0, 24, 48, and 72 h. The samples underwent centrifugation (Ortoalresa—Alvarez Redondo S.A, Madrid, Spain) at 6000 rpm for 10 min, were then filtered with Whatman filter paper, freeze-dried, and stored in the freezer at −18 °C for further analysis.

#### 3.7.3. Microbial Growth and pH Analysis

Microbial enumeration was performed to determine the viable bacterial cell counts, expressed in logarithmic colony-forming units per millilitre (log CFU/mL), using the pour plating method according to established protocols. Measurements were performed at different time intervals, as indicated above. MRS agar was used as the selective growth medium to enumerate the lactic acid bacterial strains. The fermented samples were serially diluted in sterile physiological saline solution (0.9% *w*/*v* NaCl) to achieve appropriate dilution levels for obtaining countable colonies. Aliquots from each dilution were plated in duplicate using the pour plate method, with the growth medium cooled to 45–50 °C before pouring to preserve bacterial viability. Plating procedures were carried out under aseptic conditions within a laminar flow. The inoculated plates were then incubated at 37 °C for 36–48 h to allow growth of microbial colonies before enumeration.

The pH of the fermented samples was monitored throughout the process using a calibrated pH meter Agilent Technologies 32000P (Agilent Technologies Inc., Santa Clara, CA, USA). Measurements were taken immediately after the aseptic withdrawal of the samples at each time point to track the dynamics of acidification during fermentation.

### 3.8. Monosaccharide and Organic Acid Analysis

Monosaccharides and organic acids were analysed as previously reported [57] using a Shimadzu LC-2050C3D system (Shimadzu Corporation, Kyoto, Japan) equipped with a refractive index detector. Separation was achieved on a Rezex ROA-organic acid H+ (8%) column (300 × 7.8 mm, Torrance, CA, USA), maintained at 60 °C. The mobile phase consisted of 0.005 M sulfuric acid, delivered isocratically at a flow rate of 0.5 mL/min. Qualitative determination was performed using reference materials, whereas quantitative determination was performed using external calibration curves, expressing the final concentrations as grams per litre (g/L). Details of method linearity, range, and limits of detection and quantification can be found in the Supplementary Materials (Table S3).

### 3.9. In Vitro Antioxidant Activity Assessment

#### 3.9.1. Determination of the ABTS^•+^ Scavenging Capacity

The ABTS assay was performed using the method described by Re et al. [58]. Firstly, a stock solution of ABTS^•+^ was prepared by dissolving 110 mg of ABTS and 7.6 mg of K_2_S_2_O_8_ in 100 mL of distilled water. The stock mixture was then placed in the dark at room temperature for 16 h prior to analysis. The working radical solution was then prepared by diluting the stock solution with phosphate-buffer solution until an absorbance of AU 0.700 at 734 nm was achieved. Following this, 25 μL of sample or blank was mixed with 1.5 mL of working radical solution and kept in the dark for 2 h. Then, the absorbance was measured at a wavelength of 734 nm. The antioxidant capacity was expressed as Trolox equivalent antioxidant capacity TEAC_ABTS_ (mg TE/g), calculated using a dose–response curve for Trolox.

#### 3.9.2. Determination of the Cupric Reducing Antioxidant Capacity

The CUPRAC assay was performed according to the procedure described by Apak et al. [59]: 400 μL of a sample (10 mg/mL) or blank was mixed with 400 μL of each buffer solution (ammonium acetate, neocuproine, and copper chloride). The vials were then kept in the dark for 30 min. Subsequently, the absorbance was measured at 450 nm using a GENESYS 50 UV–Vis spectrophotometer (Thermo Fisher Scientific, Waltham, MA, USA). Values were expressed as Trolox equivalent antioxidant capacity TEAC_CUPRAC_ (mg TE/g) calculated using a dose–response curve for Trolox.

#### 3.9.3. Determination of the DPPH^•^ Scavenging Assay

The DPPH assay was performed according to the method described by Brand-Williams et al. [60]. To 0.5 mL of sample or blank (methanol), 1 mL of a ~90 μmol/L (absorbance adjusted to 0.800 ± 0.010 AU at 517 nm) DPPH^•^ methanolic solution was added. Samples were then vortexed for 15 s and kept in the dark for 2 h. Following this, the absorbance was measured at 517 nm with a GENESYS 50 UV–Vis spectrophotometer (ThermoFisher Scientific, Waltham, MA, USA). Values were expressed as Trolox equivalent antioxidant capacity TEAC_DPPH_ (mg TE/g), calculated using a dose–response curve for Trolox.

### 3.10. Statistical Analysis

Mean values and standard deviations were calculated using Microsoft Excel. The statistical significance of the PLE models and individual variables was assessed using the ANOVA F-test and Student’s *t*-test (*p* < 0.05), performed with Design-Expert software version 12.0.8.0 (Stat-Ease Inc., Minneapolis, MN, USA). Model adequacy was evaluated by analysing the ‘lack of fit’ coefficient and the Fisher test value (F-value) obtained from ANOVA. For all other comparisons, one-way ANOVA followed by Tukey’s post hoc test was conducted to compare means showing significant variation (*p* < 0.05), using GraphPad Prism software version 10.5 for Windows.

## 4. Conclusions

This study further highlights the potential of *Ulva* sp. as a fast-growing green seaweed, a sustainable source of valuable bioactive compounds. The optimised PLE with dilute sulfuric acid can be seen as an efficient alternative to current pretreatment/extraction practices for recovering carbohydrates and phenolics, key ingredients with promising applications in functional foods. Under optimal PLE conditions (110 °C, three 15 min cycles, and 3.6% sulfuric acid), an extract with high yield, reducing sugar, total carbohydrate, and phenolic content was obtained. Moreover, the subsequent fermentation by *L. plantarum* confirmed the hydrolysate’s suitability as a substrate, achieving probiotic-level cell counts and lactic acid yields (~3.3 g/L) comparable to or exceeding previous reports. The chromatographic profiling provided insights into sugar utilisation patterns, revealing the complete consumption of glucose and rhamnose within 48 h, while xylose remained unfermented, highlighting the need for strain selection or co-fermentation strategies to improve substrate conversion in future studies. Nevertheless, the stability of antioxidant capacity throughout fermentation further supports the potential of this integrated approach for developing functional products, ensuring that bioactive properties are retained alongside microbial safety and functional benefits. This study demonstrates the technical feasibility of valorising *Ulva* sp. biomass through a combined PLE–fermentation strategy. However, several industrial challenges remain to be addressed, particularly the energy demands of PLE, acid neutralisation costs, and solvent recycling possibilities. In this context, comparative techno-economic and environmental assessments against enzymatic hydrolysis and other green extraction methods could further assist in validating commercial viability and scalability of the process. Also, future studies could evaluate the sensory and nutritional profiles of *Ulva*-based fermented products for food and other applications, as well as assess consumer acceptance. Additionally, exploring co-fermentation with other beneficial microbes and integrating this approach into existing food production systems may further enhance its commercial viability. Overall, this work lays the groundwork for transforming this underutilised marine resource into a valuable ingredient for future sustainable food applications.

## Figures and Tables

**Table 1:** Design matrix for PLE optimisation for extraction of *Ulva* sp. biomass and the values of observed responses: yield (g/100 g DW), TRS (mg/g E and DW), TCC (mg/g E and DW) and TPC (mg GAE/g E and DW).

Run	Independent PLE Variables			Response Factors						
	A	B	C *	RFI	RFII	RFIII	RFIV	RFV	RFVI	RFVII
	H_2_SO_4_, % (*v*/*v*)	T, °C	τ, min/cycle	Yield (g/100 g DW)	TRS (mg/g E)	TRS (mg/g DW)	TCC (mg/g E)	TCC (mg/g DW)	TPC (mg GAE/g E)	TPC (mg GAE/g DW)
1	2	90	10	30.9 ± 0.3	278.5 ± 3.5	86.1 ± 0.8	375.8 ± 1.3	116.2 ±0.5	21.9 ± 0.2	6.8 ± 0.4
2	2	90	10	32.3 ± 0.2	301.6 ± 3.4	97.4 ± 1.0	382.8 ± 1.4	123.6 ±0.6	22.5 ± 0.2	7.3 ± 0.1
3	4	120	10	48.7 ± 0.4	524.6 ± 2.5	255.3 ± 2.6	556.0 ± 5.7	270.6 ± 1.8	34.9 ± 0.4	17.0 ± 0.6
4	0	120	10	26.4 ± 0.3	249.5 ± 7.5	65.9 ± 0.8	358.5 ± 2.3	94.80 ± 0.3	17.8 ± 0.3	4.7 ± 0.0
5	2	120	15	43.7 ± 0.1	393.6 ± 2.6	172.0 ± 1.9	460.9 ± 4.8	201.4 ±1.2	28.0 ± 0.2	12.2 ± 0.45
6	4	90	5	34.1 ± 0.1	376.1 ± 2.9	128.4 ± 1.4	470.9 ± 4.9	160.7 ± 0.9	27.7 ± 0.1	9.4 ± 0.1
7	0	90	5	17.8 ± 0.4	189.3 ± 1.5	33.8 ± 0.2	293.5 ± 5.1	52.40 ± 0.2	9.6 ± 0.2	1.7 ± 0.0
8	2	90	10	30.8 ± 0.2	306.4 ± 3.5	94.5 ± 1.0	391.5 ± 1.4	120.8 ± 0.6	21.6 ± 0.1	6.7 ± 0.0
9	0	60	10	13.4 ± 0.2	172.5 ± 6.0	23.2 ± 0.1	281.3 ± 3.2	37.80 ± 0.1	6.6 ± 0.4	0.9 ± 0.0
10	2	60	5	17.6 ± 0.1	187.6 ± 6.9	33.1 ± 0.2	294.1 ± 3.6	51.80 ± 0.2	12.8 ± 0.2	2.2 ± 0.0
11	4	90	15	39.9 ± 0.2	454.6 ± 7.7	181.6 ± 2.3	520.8 ± 9.2	208.0 ± 1.3	30.5 ± 0.2	12.2 ± 0.4
12	4	60	10	30.0 ± 0.1	316.7 ± 6.0	95.1 ± 1.0	392.0 ± 3.4	117.7 ± 0.6	23.1 ± 0.2	6.9 ± 0.0
13	2	120	5	34.3 ± 0.3	353.7 ± 5.4	121.2 ± 1.2	427.1 ± 5.4	146.4 ± 0.9	24.8 ± 0.0	8.5 ± 0.1
14	2	60	15	23.6 ± 0.2	217.3 ± 6.0	51.4 ± 0.5	320.2 ± 5.2	75.70 ± 0.3	14.9 ± 0.2	3.5 ± 0.1
15	0	90	15	21.8 ± 0.4	216.7 ± 6.9	47.2 ± 0.4	315.9 ± 4.7	68.80 ± 0.2	12.0 ± 0.6	2.6 ± 0.1
16	2	90	10	32.1 ± 0.2	289.5 ± 3.4	92.9 ± 0.9	379.0 ± 1.4	121.7 ± 0.5	22.1 ± 0.2	7.1 ± 0.1
17	2	90	10	30.9 ± 0.2	293.1 ± 3.3	90.7 ± 0.94	381.9 ± 1.5	118.0 ± 0.6	21.9 ± 0.1	6.8 ± 0.2

*: Number of cycles: 3; Values are reported as mean ± standard deviation (*n* = 3). E: extract; RF: response factor; TRS: total reducing sugar content; TCC: total carbohydrate content; TPC: total phenolic content; T: temperature; τ: time.

**Table 2:** Predicted and observed values of the validation experiments performed under the suggested optimal PLE conditions.

Response Factors	Predicted Mean	95% PI Low	Experimental Value	95% PI High
RFI: Yield (g/100 g DW)	46.4	43.1	46.9 ± 0.1	49.7
RFII: TRS (mg/g E)	491.1	460.1	520.2 ± 1.9	522.1
RFIII: TRS (mg/g DW)	229.1	210.5	244.0 ± 0.9	247.7
RFIV: TCC (mg/g E)	531.7	503.4	555.1 ± 0.8	559.9
RFV: TCC (mg/g DW)	249.8	237.6	260.3 ± 0.4	262.0
RFVI: TPC (mg GAE/g E)	32.3	31.0	32.9 ± 0.1	33.6
RFVII: TPC (mg GAE/g DW)	15.2	14.1	15.4 ± 0.2	16.3

E: extract; RF: response factor; TRS: total reducing sugar; TCC: total carbohydrate content; TPC: total phenolic content.

**Table 3:** In vitro antioxidant capacity changes as measured by the CUPRAC, DPPH and ABTS assays during the fermentation of *Ulva* sp. hydrolysate.

Fermentation Time	TEAC_CUPRAC_, mg TE/g DW	TEAC_DPPH_, mg TE/g DW	TEAC_ABTS_, mg TE/g DW
0 h	49.3 ± 0.2 ^a,b^	36.9 ± 0.3 ^a^	17.11 ± 0.2 ^a^
24 h	46.0 ± 1.2 ^b^	31.7 ± 3.3 ^b^	17.01 ± 0.3 ^a^
48 h	52.5 ± 2.4 ^a^	38.5 ± 0.6 ^a^	18.0 ± 0.6 ^a^
72 h	54.2 ± 2.7 ^a^	38.3 ± 1.7 ^a^	17.6 ± 1.9 ^a^

TEAC: Trolox equivalent antioxidant capacity; TE: Trolox equivalents. Values are expressed as mean ± standard deviation (*n* = 3). Different lowercase letters within the same column indicate significant differences (one-way ANOVA, *p* < 0.05).

## Data Availability

All related data and methods are presented in this paper. Additional inquiries should be addressed to the corresponding author.

## Supplementary Materials

The following supporting information can be downloaded at https://www.mdpi.com/article/10.3390/md23100371/s1, Table S1: Analysis of variance of the regression parameters for response surface quadratic models for optimisation of *Ulva* sp. PLE extraction; Table S2: Fit statistics of the developed PLE models; Table S3: Regression parameters, linearity, LOD, and LOQ values for monosaccharide and organic acid determination; Equations (S1)–(S7): Equations in actual factors for RFI-RFVII; Figure S1: Pareto charts (*p* = 0.05), illustrating the impact of independent variables (A: sulfuric acid concentration; B: temperature, C: time) and their interactions on the response factors (RF) of *Ulva* sp. PLE optimisation.
