# Supplementary material for: Optimisation of Pressurised Liquid Extraction and Subsequent Hydrolysate Fermentation by Lactiplantibacillus plantarum for Integrated Bioprocessing of Ulva sp
Source: Mar Drugs. 2025 Sep 25;23(10):371. doi: 10.3390/md23100371 (PMC12565157; doi:10.3390/md23100371)
Supplement: Supplementary file 1 [file marinedrugs-23-00371-s001.zip › marinedrugs-3824788-supplementary.pdf]

Optimisation of Pressurised Liquid Extraction and Subsequent Hydrolysate Fermentation by *Lactiplantibacillus plantarum* for Integrated Bioprocessing of *Ulva* sp.

Aniruddh Dayanand Dave, Hakki Bilgin, Vaida Kitrytė-Syrpa, and Michail Syrpas*

Kaunas University of Technology, Department of Food Science & Technology, Radvilėnų pl. 19, LT-50254, Kaunas, Lithuania

*Corresponding Author. Email: michail.syrpas@ktu.lt; Tel: +370 (37) 30 01 88

Table S1. Analysis of variance of the regression parameters for response surface quadratic models for optimisation of *Ulva* sp. PLE extraction

Source	SS	df	MS	F-Value	p-value
**RFI: Total yield (g/100 g DW)**					
Model	1365.51	7	195.07	138.17	< 0.0001*
A-H_2SO_4, % (v/v)	671.03	1	671.03	475.28	< 0.0001*
B-Temperature, °C	584.04	1	584.04	413.66	< 0.0001*
C-Time, min/cycle	79.21	1	79.21	56.1	< 0.0001*
AB	7.95	1	7.95	5.63	0.0418*
BC	2.88	1	2.88	2.04	0.1873**
A^2	10.74	1	10.74	7.61	0.0222*
C^2	8.55	1	8.55	6.06	0.0361*
Residual	12.71	9	1.41		
Lack of Fit	10.68	5	2.14	4.22	0.0940**
Pure Error	2.03	4	0.5064		
Cor Total	1378.22	16			
**RFII: Total reducing sugar content (TRS, mg/g extract)**					
Model	1.49E+05	6	24821.37	199.73	< 0.0001*
A-H_2SO_4, % (v/v)	89042	1	89042	716.49	< 0.0001*
B-Temperature, °C	49188.16	1	49188.16	395.8	< 0.0001*
C-Time, min/cycle	3850.03	1	3850.03	30.98	0.0002*
AB	4283.7	1	4283.7	34.47	0.0002*
AC	652.8	1	652.8	5.25	0.0449*
A^2	1911.5	1	1911.5	15.38	0.0029*
Residual	1242.74	10	124.27		
Lack of Fit	770.08	6	128.35	1.09	0.4906**
Pure Error	472.67	4	118.17		
Cor Total	1.50E+05	16			
**RFIII: Total reducing sugar content (TRS, mg/g DW)**					
Model	58311.60	8	7288.95	186.22	< 0.0001*
A-H_2SO_4, % (v/v)	30049.26	1	30049.26	767.69	< 0.0001*
B-Temperature, °C	21180.94	1	21180.94	541.12	< 0.0001*
C-Time, min/cycle	2300.45	1	2300.45	58.77	< 0.0001*
AB	3451.56	1	3451.56	88.18	< 0.0001*
AC	396.01	1	396.01	10.12	0.0130*
BC	263.41	1	263.41	6.73	0.0319*
A^2	436.75	1	436.75	11.16	0.0102*
B^2	198.44	1	198.44	5.07	0.0544**
Residual	313.14	8	39.14		
Lack of Fit	240.95	4	60.24	3.34	0.1076**
Pure Error	72.19	4	18.05		
Cor Total	58624.74	16			
**RFIV: Total carbohydrate content (TCC, mg/g extract)**					
Model	98394.13	5	19678.83	166.61	< 0.0001*
A-H_2SO_4, % (v/v)	59598.78	1	59598.78	504.58	< 0.0001*
B-Temperature, °C	33140.25	1	33140.25	280.57	< 0.0001*
C-Time, min/cycle	2184.6	1	2184.6	18.5	0.0013*
AB	1883.56	1	1883.56	15.95	0.0021*
A^2	1586.93	1	1586.93	13.44	0.0037*
Residual	1299.27	11	118.12		

Lack of Fit	1161.13	7	165.88	4.8	0.0742**
Pure Error	138.14	4	34.53		
Cor Total	99693.4	16			
**RFV: Total carbohydrate content (TCC, mg/g DW)**					
Model	60383.22	8	60383.22	467.72	< 0.0001*
A-H_2SO_4, % (v/v)	31651.28	1	31651.28	1961.35	< 0.0001*
B-Temperature, °C	23134.01	1	23134.01	1433.56	< 0.0001*
C-Time, min/cycle	2541.85	1	2541.85	157.51	< 0.0001*
AB	2299.2	1	2299.2	142.48	< 0.0001*
AC	238.7	1	238.7	14.79	0.0049*
BC	241.8	1	241.8	14.98	0.0047*
A^2	210.26	1	210.26	13.03	0.0069*
C^2	79.64	1	79.64	4.94	0.0570**
Residual	129.10	8	16.14		
Lack of Fit	94.91	4	23.73	2.78	0.1733**
Pure Error	34.19	4	8.55		
Cor Total	60512.32	16			
**RFVI: Total phenolic content (TPC, mg GAE/ g extract):**					
Model	931.92	6	155.32	656.79	< 0.0001*
A-H_2SO_4, % (v/v)	616.18	1	616.18	2605.61	< 0.0001*
B-Temperature, °C	289.68	1	289.68	1224.97	< 0.0001*
C-Time, min/cycle	13.86	1	13.86	58.61	< 0.0001*
A^2	2.71	1	2.71	11.45	0.007*
B^2	1.59	1	1.59	6.72	0.0269*
C^2	6.76	1	6.76	28.57	0.0003*
Residual	2.36	10	0.2365		
Lack of Fit	1.94	6	0.3234	3.05	0.1502**
Pure Error	0.4245	4	0.1061		
Cor Total	934.28	16			
**RFVII: Total phenolic content (TPC, mg GAE/g DW):**					
Model	285.35	7	40.76	282.09	< 0.0001*
A-H_2SO_4, % (v/v)	158.78	1	158.78	1098.81	< 0.0001*
B-Temperature, °C	103.94	1	103.94	719.31	< 0.0001*
C-Time, min/cycle	9.31	1	9.31	64.41	< 0.0001*
AB	9.65	1	9.65	66.79	< 0.0001*
AC	0.8341	1	0.8341	5.77	0.0397*
BC	1.48	1	1.48	10.26	0.0108*
C^2	1.35	1	1.35	9.31	0.0138*
Residual	1.30	9	0.1445		
Lack of Fit	1.04	5	0.2086	3.24	0.1390**
Pure Error	0.2577	4	0.0644		
Cor Total	286.65	16			

*: significant; **: not significant; df: degree of freedom; F: Fisher value.; GAE: gallic acid equivalents; MS: mean square; RF: response factor; SS: sum of square.

Table S2. Fit statistics of the developed PLE models

Response factors	C.V. %	R^2	Adjusted R^2	Predicted R^2
RFI: Total yield (g/100 g DW)	3.97	0.9908	0.9836	0.9412
RFII: TRS, mg/g E	3.70	0.9917	0.9868	0.9719
RFIII: TRS, mg/g DW	6.37	0.9947	0.9893	0.9603
RFIV: TCC, mg/g E	2.80	0.9870	0.9810	0.9500
RFV: TCC, mg/g DW	3.27	0.9979	0.9957	0.9848
RFVI: TPC, mg GAE/g E	2.34	0.9975	0.9960	0.9910
RFVII: TPC, mg GAE/g DW	5.54	0.9955	0.9919	0.9780

E: extract; GAE: gallic acid equivalents; TRS: total reducing sugar content; TCC: total carbohydrate content; TPC: total phenolic content

Table S3. Regression parameters, linearity, LOD, and LOQ values for monosaccharide and organic acid determination

Compound	Calibration equation	R^2	LOD (g/L)	LOQ (g/L)	Range (g/L)
Lactic acid	$y = 63740x - 6516$	0.9981	0.026	0.078	0.1–5.0
Acetic acid	$y = 54906x - 748$	0.9975	0.030	0.091	0.1–5.0
Glucose	$y = 119917x - 3817$	0.9992	0.014	0.042	0.1–5.0
Xylose	$y = 124953x - 3962$	0.9989	0.013	0.040	0.1–5.0
Rhamnose/arabinose	$y = 122487x - 3923$	0.9987	0.014	0.041	0.1–5.0

LOD: limit of detection; LOQ: limit of quantification

Equations S1-S7: Equations in actual factors for RFI-RFVII

$$\textbf{RFI} = -7.71838 + (4.06013*A) + (0.1813*B) + (1.25902*C) + (0.02349*AB) + (0.00565283*BC) + (-0.398741*A^2) + (-0.0569236*C^2) \quad \textbf{(Eq. S1)}$$

$$\textbf{RFII} = 51.6125 + (-30.35694*A) + (1.52292*B) + (1.8325*C) +(0.545417*AB) + (1.2775*AC) + (5.31111*A^2) \quad \textbf{(Eq. S2)}$$

$$\textbf{RFIII} = 69.23179 + (-33.53928*A) + (-1.17611*B) + -(3.4675*C) + (0.489583*AB) + (0.995*AC) + (0.0541*BC) + (2.54263*A^2) + (0.007617*B^2) \quad \textbf{(Eq. S3)}$$

$$\textbf{RFIV} = 151.2625 + (-8.75069*A) + (1.42208*B) + (3.305*C) + (0.361667*AB) + (4.83924*A^2) \quad \textbf{(Eq. S4)}$$

$$\textbf{RFV} = -14.70105 + (-19.29434*A) + (0.475*B) + (0.829526*C) + (0.399583*AB) + (0.7725*AC) + (0.0518333*BC) + (1.76421*A^2) + (-0.173726*C^2) \quad \textbf{(Eq. S5)}$$

$$\textbf{RFVI}= -18.8323 + (5.18988*A) + (0.323433*B) + (1.27665*C) + (-0.200438*A^2) + (-0.0006825*B^2) + (-0.05067*C^2) \quad \textbf{(Eq. S6)}$$

$$\textbf{RFVII}= -3.32786 + (-0.559089*A) + (0.027793*B) + (0.210042*C) + (0.025889*AB) + (0.045666*AC) + (0.004058*BC) + (-0.022545*C^2) \quad \textbf{(Eq. S7)}$$

Where: A: sulfuric acid concentration (0-4%, v/v), B: temperature (60-120 °C), C: time (3 cycles of 5-15 min/cycle); RFI: Total yield (g/100 g DW); RFII: Total reducing sugar content (TRS, mg/g extract); RFIII: Total reducing sugar content (TRS, mg/g DW); RFIV: Total carbohydrate content (TCC, mg/g extract); RFV: Total carbohydrate content (TCC, mg/g DW); RFVI: Total phenolic content (TPC, mg GAE/g extract); RF VII: Total phenolic content (TPC, mg GAE/g DW)

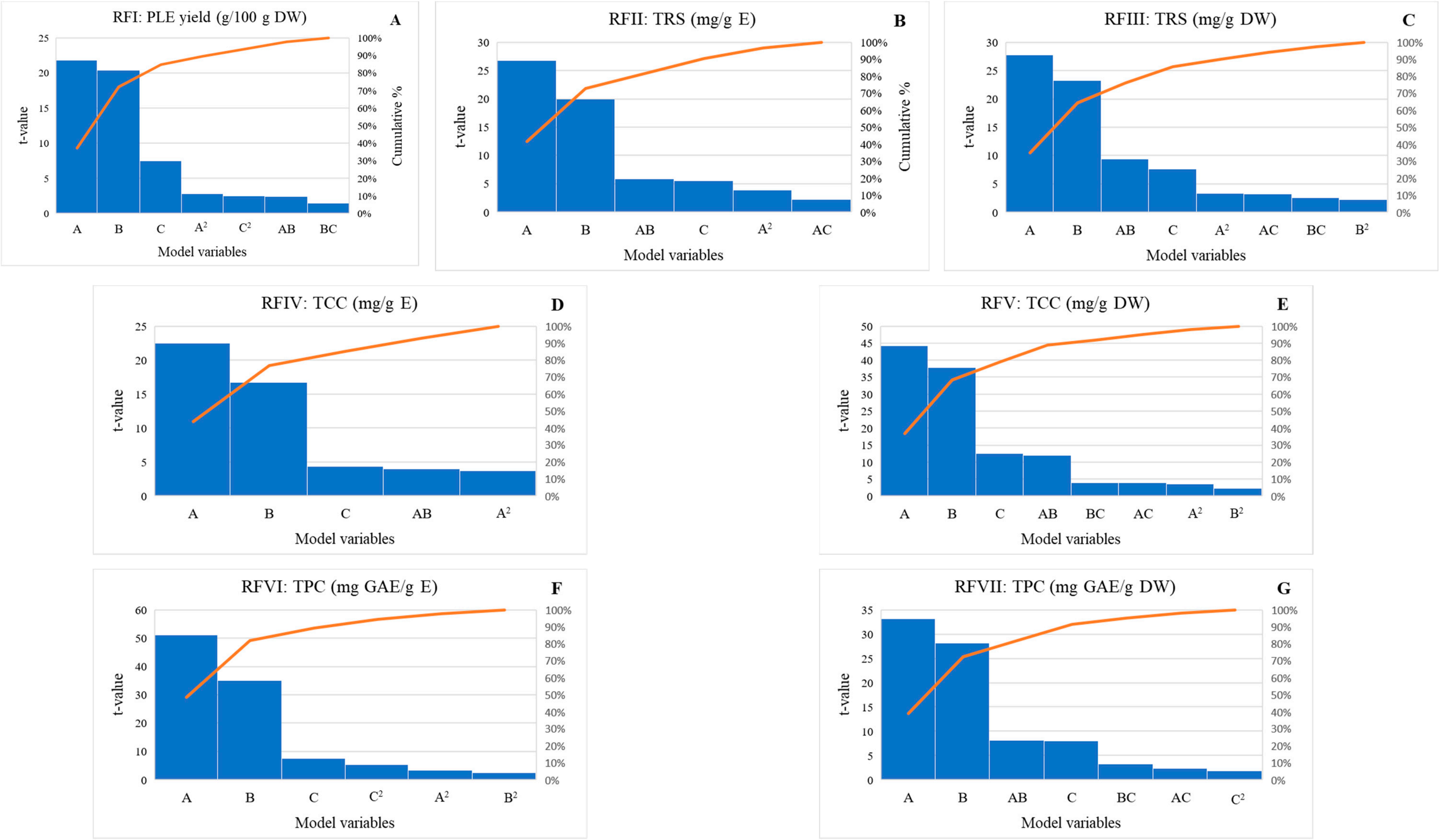

Fig. S1. Pareto charts ($p < 0.05$), illustrating the impact of independent variables (A: sulfuric acid concentration; B: temperature, C: time) and their interactions on the response factors (RF) of *Ulva* sp. PLE optimisation
